# Intruder traits modulate aggressive behavior of territory owners

**DOI:** 10.1038/s41598-020-68513-1

**Published:** 2020-07-21

**Authors:** Caio Akira Miyai, Fábio Henrique Carretero Sanches, Tânia Márcia Costa, Rodrigo Egydio Barreto

**Affiliations:** 1 Aquaculture Center, CAUNESP, São Paulo State University, UNESP, Jaboticabal, SP 14884-900 Brazil; 2 Biosciences Institute, São Paulo State University (UNESP), Coastal Campus, Pça. Infante D. Henrique s/n, São Vicente, SP 11330-900 Brazil; 3 Institute of Marine Sciences, Federal University of São Paulo - IMar/UNIFESP, Rua Dr. Carvalho de Mendonça, 144, 11070-100 Santos, São Paulo Brazil; 4 Department of Structural and Functional Biology, Institute of Biosciences of Botucatu, UNESP, R. Prof. Dr. Antônio Celso Wagner Zanin, 250 - Distrito de Rubião Junior – Botucatu, 18618-689 São Paulo State Brazil

**Keywords:** Animal behaviour, Animal physiology

## Abstract

The prior residence effect consists of a higher probability of territory owners to win a fight against intruders, as observed in several territorial species across animal kingdom. However, in investigations on territorial behavior, intruder traits have been assumed as fixed. Thus, we used a territorial species as experimental model to evaluate the effect of intruder traits on aggressive behavior of territory owners. During fights staged between territory owners and intruders, intruder traits, linked with visual signals of social status (dominant-subordinate position), modulate fighting behavior of territory owners, but prior residence effect still occurred. Intruder traits must be addressed more effectively for improving the theoretical understanding of territoriality.

A territory is an area in which an animal (a territory owner) maintain sole or prioritized access to space and other resources it encompass^1,2^. In this context, aggressive behavior in defense of a territory against intruders may take place, a component of territoriality^3–6^. Owning a territory demands costs in terms of fitness benefits because owner animals spend energy and time to search for and to combat off territory intruders^7^. Nevertheless, the costs to keep a territory must be lower than the benefit of having exclusive access to territory resources to justify the owners engaging in fights against intruders^8^ and these costs could be associated to opponent quality. Thus, adjustment in aggressive activities by owners could be adaptive depending on the intruder traits. However, studies that investigate territoriality have assumed intruder traits as fixed. At most, in experimental approaches, intruders have been putatively considered as non-territorial floaters or territory neighbors, aiming to obtain the territory, or expanding territory borders and/or stealing specific resources^2^. Therefore, we hypothesized that intruder traits influence the aggressive behavior of resident animals.

In social hierarchies, social status can be signaled to maintain stable social structures, because fighting against dominant individuals tends to be avoided^9,10^. In this sense, dominance signals could be considered as potential traits of being under evaluation of territory owners before engaging in a fight and/or dealing with an intruder competitor. Dominance signals could indicate fighting motivation/ability, especially because dominant animals tend to win and subordinate to lose future fights^11^ and, in fact, the opponent social rank position can be inferred by visual inspection in animals^12^. Regarding the resident-intruder experimental paradigm, we staged fights between territory owners and intruders that exhibited signals of social hierarchy positions. As experimental animal model, we used an aggressive species with clear territorial behavior and dominant-subordinate status signaled via visual cues: the fish pearl cichlid, *Geophagus brasiliensis*^13,14^. Specifically, we tested whether the body color of an intruder pearl cichlid affects the aggressive behavior of a resident fish, since in some cichlids species subordinate status is usually associated with the appearance of dark stripes, whereas dominant fish are typically paler^15,16^.

This research agrees with the Ethical Principles in Animal Research adopted by the National Council for the Control of Animal Experimentation—Brazil (CONCEA—Conselho Nacionalde Controle de Experimentação Animal—Brazil) and was approved by the CEUA—Ethical Committee for Animal Research (CEUA—Comissão de Ética no Uso de Animais—Protocol 1153) from the Institute of Bioscience of Botucatu/UNESP (Instituto de Biociências de Botucatu/UNESP). No fish got seriously injured or died due to the agonistic interactions. Additionally, at the end of the experiments, all individuals were maintained in recovery tanks (120 L; 70 × 35 × 50 cm; with a holding density of one fish per 1.5 L of water), kept for two weeks, and released at the site where they were collected.

To conduct our experiment, we captured 48 fish from glass holding tanks (see Supplementary information) and placed them into glass aquaria (40 × 20 × 25 cm) in social isolation (one fish per aquarium) for 5 consecutive days to induce a prior residence condition^14^. We observed 48 encounters between body color-modulated and non-modulated fish of equal size. Of these 48 pairs, 24 resident fish were paired with body color-modulated intruder fish in their residence aquaria. As control of prior residence effects, the other 24 fish were paired with body color-modulated fish in neutral arenas. The body color-modulated fish could be dark-striped, pale, or have an intermediate body color pattern. The color of non-modulated fish was similar to intermediate pattern (Fig. 1). We had a total of six independent conditions (n = 8 pairs each). The paired individuals were of similar size (Supplementary Table 1), with any difference not exceeding 3.2% (mean ± SD = 0.62 ± 0.70%; minimum = 0.0% and maximum = 3.2%). A size difference within this range avoids asymmetric body size effects (up to 10%)^17^, but we also confirmed the absence of effects of body size in winning a fight (see Supplementary information).

**Figure 1:**
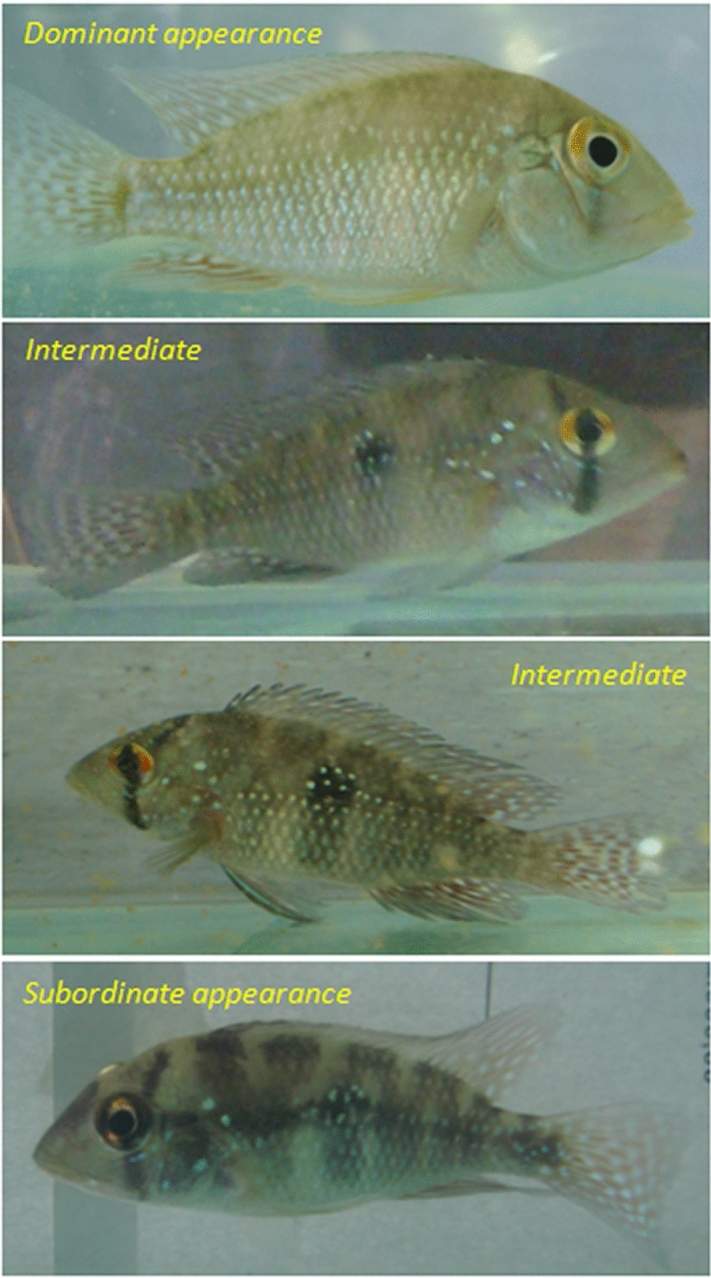
Modulation of body color in the pearl cichlid, *Geophagus brasiliensis.* These body color patterns were reached by keeping them in tanks with monochromatic backgrounds: white background for dominant appearance (a pale pattern), blue background for an intermediate pattern, and black background for a subordinate appearance (a dark-striped pattern). Fish with non-modulated body color were kept in glass holding tanks, displaying an intermediate pattern.

For pairing, fish were caught from their respective aquaria using a net and introduced into the opponent resident fish’s aquaria or a neutral arena. To control handling disturbance, the resident fish were also captured and reintroduced into their own aquarium simultaneously with the intruder fish, while in the neutral arena both fish were introduced at the same time into the neutral aquarium. As dominance hierarchy is quickly defined in pearl cichlids^14^ and in other cichlid species^18^, the paired fish were video-recorded for 10 min. This time was enough for fishes to stablish stable dominance, with no visible injuries to the submissive fish. The total number of attacks was quantified from these videos.

We quantified the directed attacks by counting the incidences of biting on the tail fin, anterior (head), median, and ventral areas, lateral fighting (a sudden slap between fish bodies), chasing, and mouth wrestling. The initiator of an attack was identified by observing who approached the opponent and directed the attack. The loser was the fish that left the place of attack or retreated first. The dominant and subordinate animals were identified by calculating the dominance index (DI) according to Bailey et al.^19^. The DI values range from 0 to 1, and the greater the DI value, the higher the animal’s rank in regard to dominance. Dominant fish had DI value greater than 0.5^19,20^. The DIs of body color non-modulated fish are presented in our Supplementary spreadsheet.

For changing fish body color, they were isolated in stocking aquaria for at least 30 days before testing (~ 70 L; 60 × 35 × 35 cm; 1 fish/1.5 L of water). Aquaria had walls of white, black, or blue colors. This process induces body color changes in pearl cichlid, becoming them pale, dark-striped, or an intermediate pattern (varying between the pale and the dark profiles, but not achieving either), respectively. Fish with non-modulated body color were kept in glass holding tanks, displaying an intermediate pattern (Fig. 1). This effect lasts several days, and this procedure did not induce stress (indicated by plasma cortisol levels—see Supplementary information).

Normality and homoscedasticity were confirmed by the Kolmogorov–Smirnov test and the Levene test, respectively. To assure that a prior residence effect occurred, we pooled the frequency of dominant and subordinate fish in each condition (prior residence or neutral arena) and compared these frequencies by using two-sided Fisher’s exact test. To confirm the absence of effect of the procedures for body color changes on aggressiveness, we compared attack rates of body color modulated fish among conditions by two-way ANOVA, having territorial ownership condition (prior residence × neutral arena) and body color (pale, dark-striped or intermediated) as factors. To evaluate the specific effect of opponent body color on aggression, we compared the attack rate between body color non-modulated and modulated fish of each independent condition by two-tailed paired Student’s t-test. A difference was considered significant when P < 0.05.

Our procedures to induce longstanding body color changes did not affect aggressiveness (Two-way ANOVA effects; prior residence/neutral arena factor, F_(1;42)_ = 1.25, P = 0.27; body color factor, F_(1;42)_ = 0.69, P = 0.51; interaction, F_(1;42)_ = 0.18, P = 0.84; Fig. 2).

**Figure 2:**
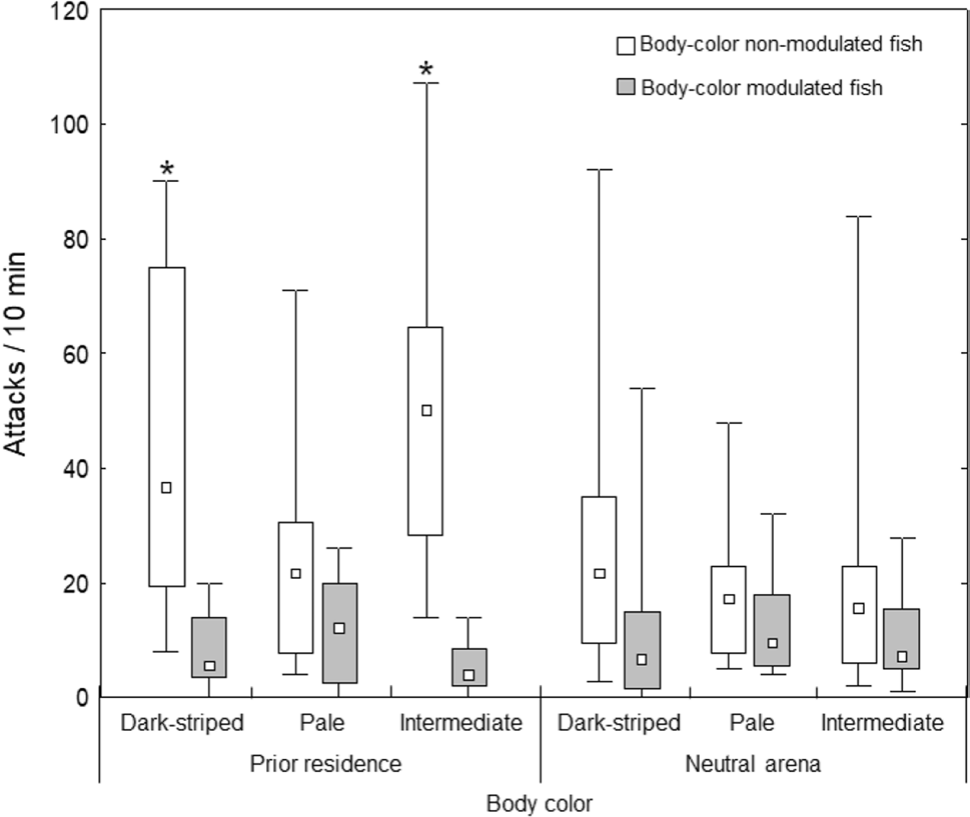
The effects of body color pattern and prior residence on attack rate in pearl cichlids. Asterisk indicates a statistical difference between attack rates (the small white square are medians, boxplots represent 25–75% interquartile range and whiskers are minimum and maximum values for each boxplot) obtained for body color modulated and non-modulated fish within the same opponent body color condition (dark-striped, pale, or intermediate) in a same territorial ownership condition (prior residence or neutral arena).

In relation to the encounters between body color modulated and non-modulated fish, when in prior residence, 22/24 resident non-modulated fish and 2/24 intruder color-modulated fish became the dominant fish. Whilst in the neutral arena (control condition), 14/24 non-modulated fish and 10/24 modulated ones became the dominant fish, indicating a significant prior-residence effect (two-sided Fisher’s exact test; P = 0.017).

In respect of directed attack between opponents (Fig. 2), we found that, when in prior residence, residents (body color non-modulated fish) attacked more dark-striped intruders (two-tailed paired Student t test; t_7_ = 3.9; P = 0.006) and those fish with intermediate body color (t_7_ = 2.8; P = 0.025). When intruders had pale bodies, the number of directed attacks by resident animals against intruders was statistically indistinguishable (t_7_ = 1.5; P = 0.17). In neutral arena, the number of directed attacks by body color non-modulated fish were similar to the number of their body-color manipulated opponents (dark-striped opponents, t_7_ = 1.2; P = 0.27; intermediate opponents, t_7_ = 0.9; P = 0.37; pale opponents, t_7_ = 1.0; P = 0.34).

The probability of a resident fish wins a fight, becoming the dominant fish was clear in our observations, confirming a prior resident effect^14,17,21–24^. This effect is not observed only in fish species, but across animal kingdom, in taxa such as crustaceans^25^, echinoderms^6^, amphibians^26^, birds^27^ and mammals^28^. The findings herein validate our analysis in the context of the resident-intruder interactions. In a general manner, territorial intruder traits affect resident animal aggressive behavior, indicating that territorial defense is not a result of unilateral process and, for a better understanding of the evolution of territorial behavior, intruder traits must be more extensively considered. Here, specifically, body colors of intruder fish affect aggressive behavior of resident fish. For pearl cichlid, a pale appearance of an intruder animal (a dominant visual signal) induced similar attack rate by resident fish against them, whilst resident fish attacked significantly more the dark and the intermediate intruders than received attacks from these respective intruder opponents (Fig. 2). In other words, intruder fish with dark-striped pattern (a subordinate signal) or social-rank undefined visual signal were more attacked by resident animals than directed attack against them.

In relation to the contests carried out in the neutral arena, no advantage was found by any fish (body color-modulated or not). In fact, both fish in the pair were intruders in relation to the pairing territory and the tested fish in this condition had no other asymmetry that could confer an advantage in the fight, such as a larger body size^21,29,30^ or a history of previous dominance^11,30^. Therefore, when establishing a new territory, to obtain ownership should be more relevant irrespective of opponent body color (i.e. opponent signaling status).

The behavior of resident animal against the opponent can be explained in terms of probability to engage in a costly combat. Dark-striped intruders display a subordinate signal and could be interpreted as poor competitors leading to an increase in residents aggression to defend their territories. A similar outcome was found to intermediate body color pattern, a typical condition before the fight resolution and dominance-subordination establishment and status signaling. The resident fish fiercely attacked the intruders in these cases, an observation that is in accordance to the pay-off asymmetry between resident and intruder, in which resident fish is supposed to value its territory more than an intruder and tend to defend it more. However, fighting off an intruder with pale dominant appearance, a trait that can represent a good competitor, possibly indicates an engagement in a disadvantageous combat. In fact, in a related species, the African cichlid fish (*Astatotilapia burtoni*), dominant males increase chemical signals (via increased urination rate) during territorial behaviors when exposed to another dominant male to ostensibly communicate information on dominance status^31^.

In conclusion, intruder traits modulate fighting behavior of territory owners, while in neutral arenas both competitors do not own the territory, showing that, ownership should be more relevant in those cases. Our findings evidence the need to consider resident, but also intruder traits in order to better comprehend the territoriality and the prior residence paradigm.

## Supplementary information

Supplementary data

Supplementary information
