# Supplementary material for: Intruder traits modulate aggressive behavior of territory owners
Source: Sci Rep. 2020 Jul 21;10:12050. doi: 10.1038/s41598-020-68513-1 (PMC7374745; doi:10.1038/s41598-020-68513-1)
Supplement: Supplementary file 2 — Supplementary information [file 41598_2020_68513_MOESM2_ESM.docx]

**Intruder traits modulate aggressive behavior of territory owners**

Caio Akira Miyai^1,2^, Fábio Henrique Carretero Sanches^2,3^, Tânia Márcia Costa^1,2^ and Rodrigo Egydio Barreto^1,4^ *

^1^ Aquaculture Center - CAUNESP, São Paulo State University - UNESP, 14884-900, Jaboticabal, SP, Brazil.

^2^ Biosciences Institute, São Paulo State University (UNESP) – Coastal Campus, Pça. Infante D. Henrique s/n, 11330-900, São Vicente, SP, Brazil.

^3^ Institute of Marine Sciences, Federal University of São Paulo - IMar/UNIFESP, Rua Dr. Carvalho de Mendonça, 144, 11070-100, Santos, São Paulo, Brazil.

^4^ Department of Structural and Functional Biology, Institute of Biosciences of Botucatu, UNESP, R. Prof. Dr. Antônio Celso Wagner Zanin, 250 - Distrito de Rubião Junior – Botucatu, 18618-689, São Paulo State, Brazil

* Corresponding author e-mail: rodrigo.egydio@unesp.br

Phone: 55-14-3880-0327

**Animals and holding conditions**

Specimens of the pearl cichlid, *Geophagus brasiliensis*, (Quoy and Gaimard, 1824) were collected from a lagoon system in a rural area of São Paulo, district of Jardim Britânia (23°25’46.34’’S, 46°47’19.47’’W), SP, Brazil. The fish were of both sexes and were sexually immature (7.13 ± 0.63 cm). Fish were held in four indoor glass tanks (120 L; 70 × 35 × 50 cm) with a holding density of one fish per 1.5 L of water. This stock population was held for at least 1 month prior to testing. During this time, the water was kept at a mean temperature of 25 ± 1 °C, with biological, chemical, and mechanical filtering and constant aeration with an air pump (air stone connected via plastic tubing). Approximately 20% of the tank water was slowly changed at least three times per week (dechlorinated water). Ammonia and nitrite levels were <0.05 ppm and <0.5 ppm, respectively. The photoperiod was ~12 h light and ~12 h dark. Fish were fed daily with a commercial fish feed containing 32% protein (Presence; Evialis do Brasil Nutrição Animal, Paulínia, SP, Brasil).

**Supplementary Table 1 – Mean (± SD) of standard body length (cm) of tested pearl cichlids, *Geophagus brasiliensis*.**

| Body color pattern | Prior residence | | Neutral arena | |
| --- | --- | --- | --- | --- |
|  | Body color modulation | | | |
|  | Non-modulated | Modulated | Non-modulated | Modulated |
| Dark-striped | 7.0 ± 0.4 | 7.0 ± 0.3 | 6.9 ± 0.1 | 6.9 ± 0.1 |
| Pale | 7.0 ± 0.2 | 6.9 ± 0.2 | 7.1 ± 0.4 | 7.1 ± 0.4 |
| Intermediate | 7.3 ± 0.2 | 7.2 ± 0.2 | 7.0 ± 0.3 | 7.0 ± 0.3 |

In the present study, a body color-modulated fish was paired with a non-modulated fish either in a prior residency or a neutral arena territorial condition. The body color of the modulated fish was reached by keeping them in tanks with monochromatic backgrounds: black background for a dark-striped pattern, white background for a pale pattern, and blue background for an intermediate pattern (Fig. 1). The size of the fish were not statistically different between these six conditions (two-way ANOVA with repeated measures two-way; F_(2;42)_ = 0.960; P = 0.391; n = 8 pairs each condition).

**Effects of body size on fight resolution**

We compared the size of the winner and the loser fish using a mixed model ANOVA, having territorial ownership as an independent factor and contest resolution (the winner or the loser fish in the pair) as repeated measures. The winning fish were not significantly larger than the losing fish (F_(1;46)_ = 1.693; P = 0.200; mean (± SD) length (cm): prior residency, winner length = 7.10 ± 0.28 and loser length = 7.08 ± 0.28; neutral arena, winner length = 6.92 ± 0.33 and loser length = 6.93 ± 0.38. Thus, we concluded that the outcome of the contests was not influenced by a body size effect.

**Effects of tank color in plasma cortisol levels**

We confirm that plasma cortisol levels were similar among fish reared in tanks with white, black or blue walls (one-way ANOVA; F(2;33) = 0.33; P = 0.72; mean (± SD; n = 12) cortisol (ng/mL): white = 124 ± 83; black = 100 ± 60; blue = 116 ± 66). These values were similar to resting values showed previously for this species^1^. Holding and water conditions were as described above for the stock population (see above).

*Plasma cortisol analyses*

We analyzed plasma levels of cortisol from blood samples. Each fish was gently removed from the stocking aquaria (white, black or blue walls) and anaesthetized with benzocaine (80 mg/L). After partial anesthesia^2^, blood was collected by cardiac puncture using heparinized insulin syringes (1 mL) and centrifuged at 754.65 RCF (g) for 10 min, in which plasma was collected and stored frozen at −20 °C. To determine the plasma cortisol levels, we used a commercial enzyme-linked immunosorbent assay ELISA kit (DRG^®^, International Inc., Cortisol Enzyme Immunoassay Kit). The ELISA assay is considered a precise and accurate assessment of cortisol levels in teleost fish^3,4,5^, including other cichlids^6,7,8^.

^1^Batista, L. Estresse crônico prejudica aprendizado em peixes (*Geophagus* *brasiliensis*). MSc Thesis UFPR (2013). https://acervodigital.ufpr.br/handle/1884/32266

^2^Gontijo A.M.M.C., Barreto R.E., Speit G., Reyes V.A.V., Volpato G.L. & Salvadori D.M.F. Anesthesia of fish with benzocaine does not interfere with comet assay results, *Mutat. Res. Toxicol. Environ. Mutagen.* **534**, 165–172(2003), http://dx.doi.org/10. 1016/S1383-5718(02)00276-0.

^3^Sink T.D., Lochmann R.T. & Fecteau K.A. Validation, use, and disadvantages of en- zyme-linked immunosorbent assay kits for detection of cortisol in channel catfish, largemouth bass, red pacu, and golden shiners, *Fish Physiol. Biochem*. **34**, 95–101 (2008). http://dx.doi.org/10.1007/s10695-007-9150-9.

^4^Barry T.P., Lapp A.F., Kayes T.B. & Malison J.A. Validation of a microtitre plate ELISA for measuring cortisol in fish and comparison of stress responses of rainbow trout (*Oncorhynchus* *mykiss*) and lake trout (*Salvelinus* *namaycush*). *Aquaculture* **117**, 351–363 (1993). http://dx.doi.org/10.1016/0044-8486(93)90331-R.

^5^Lupica S.J. & Turner J.W. Validation ofenzyme-linked immunosorbent assay for mea- surement of faecal cortisol in fish. *Aqua*. *Res*. **40**, 437–441 (2009). http://dx.doi.org/ 10.1111/j.1365-2109.2008.02112.x.

^6^Welker T.L., Lim C., Yildirim-Aksoy M. & Klesius P.H. Growth, immune function, and disease and stress resistance of juvenile Nile tilapia (*Oreochromis* *niloticus*) fed grad- ed levels of bovine lactoferrin. *Aquaculture* **262**, 156–162 (2007). http://dx.doi.org/ 10.1016/j.aquaculture.2006.09.036.

^7^Miyai, C.A., Sanches, F.H.C., Pinho-Neto, C.F., Barreto, R.E. Effects of predator odor on antipredator responses of Nile tilapia. *Physiol*. *Behav*. **165**, 22–27 (2016). <https://doi.org/10.1016/j.physbeh.2016.06.033>

^8^Sanches, F.H.C., Miyai, C.A., Pinho-Neto, C.F., Barreto, R.E., 2015. Stress responses to chemical alarm cues in Nile tilapia. Physiology & Behavior 149, 8–13. https://doi.org/10.1016/j.physbeh.2015.05.010
